# Adaptive Diagnosis for Fault Tolerant Data Fusion Based on *α*-Rényi Divergence Strategy for Vehicle Localization

**DOI:** 10.3390/e23040463

**Published:** 2021-04-14

**Authors:** Khoder Makkawi, Nourdine Ait-Tmazirte, Maan El Badaoui El Najjar, Nazih Moubayed

**Affiliations:** 1 CRIStAL, Centre de Recherche en Informatique Signal et Automatique de Lille, Université de Lille, CNRS, UMR 9189, F-59000 Lille, France; maan.el-badaoui-el-najjar@univ-lille.fr; 2 Azm Center for Research in Biotechnology and Its Application, EDST, Lebanese University, Tripoli 1300, Lebanon; 3 IFSTTAR, COSYS, LEOST, Université Gustave Eiffel, F-59650 Villeneuve D’Ascq, France; nourdine.ait-tmazirte@univ-eiffel.fr; 4 CRSI LaRGES, Lebanese University, Tripoli 1300, Lebanon; nmoubayed@ieee.org

**Keywords:** adaptive diagnostic, multi-sensor fusion, fault detection and isolation, α-Rényi Divergence, adaptive thresholding, localization, GNSS

## Abstract

When applying a diagnostic technique to complex systems, whose dynamics, constraints, and environment evolve over time, being able to re-evaluate the residuals that are capable of detecting defaults and proposing the most appropriate ones can quickly prove to make sense. For this purpose, the concept of adaptive diagnosis is introduced. In this work, the contributions of information theory are investigated in order to propose a Fault-Tolerant multi-sensor data fusion framework. This work is part of studies proposing an architecture combining a stochastic filter for state estimation with a diagnostic layer with the aim of proposing a safe and accurate state estimation from potentially inconsistent or erroneous sensors measurements. From the design of the residuals, using α-Rényi Divergence (α-RD), to the optimization of the decision threshold, through the establishment of a function that is dedicated to the choice of α at each moment, we detail each step of the proposed automated decision-support framework. We also dwell on: (1) the consequences of the degree of freedom provided by this α parameter and on (2) the application-dictated policy to design the α tuning function playing on the overall performance of the system (detection rate, false alarms, and missed detection rates). Finally, we present a real application case on which this framework has been tested. The problem of multi-sensor localization, integrating sensors whose operating range is variable according to the environment crossed, is a case study to illustrate the contributions of such an approach and show the performance.

## 1. Introduction

More than 90 per cent of road crashes are the result of driver error [1], causing millions of people die from traffic accidents worldwide each year, according to a study by the U.S. Department of Transportation’s National Highway Safety Administration. While other factors often come into play, such as distractions due to weather and/or other vehicles, and physical limitations, such as vision and response time. These reasons are creating great interest in providing safe driving technologies. Starting by helping the human driver through an Advanced Driver Assistance Systems (ADAS), toward a full automated vehicle. The functions to be developed to replace the driver are numerous. However, to simplify, let us retain four main functions:

- Localization: being the ability to know how to define more or less precisely, and in an absolute or relative way, its position;
- Perception: knowing how to analyze the nearby environment and act accordingly (detection of obstacles, signaling, etc.);
- Control: take control of the vehicle’s actuators (acceleration, braking, steering angle, etc.); and,
- Navigation: knowing how to plan and execute a route to a destination.

All of these functions are interdependent and they must meet the same requirements: the robustness, availability, accuracy, reliability, and of course safety for the surrounding and the system itself. The localization function is particularly interesting insofar as it provides information considered to be critical from a safety point of view and used as input by the other functions. The Global Navigation Satellites System (GNSS) is the most commonly used localization system for autonomous vehicles, as it is cheap and easily accessible. It also provides a first position without prior knowledge. However, GNSS standalone suffers, in land applications, from poor reliability due to limitation in precision, which affects the safety-critical Intelligent Transportation System (ITS) [2]. Different techniques for enhancing the performance of GNSS exist in the literature. In [3] and [4], the map-matching algorithm is applied. One of the limits of this technique is that the performance of a map-matching algorithm depends strongly on the resolution of the digital map. As well, the processing time for the estimation is typically high [2].

Based on the sensors fusion technique, a GNSS and Inertial Measurement Unit (IMU) sensor fusion approach provides a centimeter level of accuracy under no GNSS signal loss condition [5]. When the GNSS signal is lost for three seconds, the performance obviously decreases to a meter level. For better performance, [6] uses cameras for lateral positioning based on lane markers recognition with the GNSS/IMU for global positioning. To decrease the cost of used sensors, [7] rely on a low cost pulse-based Short Range Radar (SRR) and were able to achieve a good accuracy in their application. Additionally, in [8], GNSS, IMU, and odometer are integrated, and an infrared LiDAR is used to generate high-resolution maps. Subsequently, a SLAM relaxation algorithm that is based on PF localizes the vehicle in the created map. The two previous approaches offer good performance with an acceptable mean errors, but the drawback of the LiDAR, concerning the power needed, the computational requirements and its implementation costs, is considered high.

As we have seen, sensors are indispensable elements in ITS, even if they have limitations. However, these limitations create faults that directly affect the system performance. Hence, there must be a way to monitor the system and take an appropriate decision in the case of faults’ existence. Therefore, problems and faults due to feared events can be detected and corrected while the system is still available and safe. This avoids future failures in the system as well as costs that result from repairs. In navigation systems, detecting the fault is not enough to ensure the availability; thus, the fault must be monitored and evaluated in order to isolate or identify the error. Advanced methods of diagnostic, Fault Tolerance (FT), and fault management, called Fault Detection and Isolation (FDI) [9], are valuable tools for satisfying such needs. The existing approaches in the literature are based on the duplication and comparison techniques that can be divided into two categories [10]: Hardware Redundancy approaches and Analytical Redundancy approaches.

The basic concept of the hardware redundancy approaches is to measure a single crucial input parameter with two or more sensors (usually three or more), and then the operation of detection is done through consistency checking provided by the redundant sensor measurements, and the isolation for the faulty sensor using majority voting process. In [11], an FDI method is presented and a feasibility study for fault detection is shown by the temporal analysis of conflict resulting from combining three data sources that are based on Smets’s Transferable Belief Model (TBM). Accordingly, if the conflict of the source is high when compared to the other sources, it is considered to be faulty and it is isolated before the final fusion process. An adaptive technique to weight the outputs sensors is presented in [12]. Where, authors estimates the standard deviation of each sensor by statistics and a time factor related to the previous data, then mitigate the impact of of hardware degradation by calculating the sensor output update factor for the data fusion process. In [13], data fusion approaches are described in order to increase the accuracy and improve the fault tolerance of inertial network systems. These approaches are based on the Kalman Filter (KF) techniques for the fusion process, and use the IMU redundancy for fault masking, taking one master node for the navigation states estimation and slave nodes for the local states and local inertial vector information.

On the other hand, the concept of the analytical redundancy approaches (also known as functional, inherent, or artificial redundancy) is achieved by finding the relation between the measured inputs based on mathematical model, and generates residuals in order to detect and isolate the faulty sensor [14]. After the residuals generation, a decision is made through thresholding technique to evaluate the residuals and make the final decision for detection and isolation. For example, based on the innovation that is calculated using the KF, [15] describes a general approach for error detection, diagnosis, and prognosis in systems that can be described using mathematical models. It is based on System Theory and Statistical Decision Theory. The paper considers the special case of Gaussian random input linear dynamic systems and shows how the statistical properties of the innovation process can be used for error detection and diagnosis. A Fault Detection (FD) algorithm, based on the Extended Kalman Filter (EKF) to track the outputs from the localization methods, followed by the CUmulative SUM (CUSUM) to test the filter’s residual, is proposed in [16] to identify any unexpected large deviation. The authors in [17] propose an FD method for a woodland vehicle localization. Data fusion occurs using GPS, encoders, and IMU under EKF estimator after a Normalized Innovation Squared (NIS) tests dealing swith GPS measurements.

In all of the articles cited above, the environment in which the system operates is always considered the same, which directly affects the way in which the limitations of the sensors are treated in the diagnostic part. Where, in fact, these limitations are related to different elements that depend on the type of application and the surrounding environment of the system. In this paper, the objective is to develop an Adaptive Fault-Tolerant Fusion (AFTF) localization approaches for autonomous vehicles by multi-sensor data fusion, through the integration of a diagnostic layer that allows the detection and isolation of faulty sensors. More specifically, we are interested in the development of systems to provide a measure of confidence in the calculated information and improve the accuracy of the positioning system. Where, in the diagnostic part, our developments are oriented towards the use of informational approaches based on the use of filtering techniques and informational metrics for residuals generation. These residuals can be created by different existing metrics. Starting by the euclidean metric, also called euclidean distance [18], which is considered to be the simplest way to compare the two distributions using the distance between their means. But taking only the mean as comparison factor leads to a big lose in information related to the characteristics of the distributions, such the volume, the form and the orientation. In 1948, Claude Elwood Shannon proposed [19] a new concept dealing with information, called Shannon’s entropy. This information theory allows for generating residuals while taking the means as well as the uncertainty into consideration as an important factor. Recently, researchers resorted involving information metrics as support for the design in the residuals in FDI methods in order to reach higher integrity and accuracy through FDI methods. The Mutual Information (MI) in [20] and [21] is used as similarity measure for the residual created and it shows higher accuracy and better results when comparing with other methods. In [22], a robust unknown input observer for a class of nonlinear systems is designed for FDI methods through the Bhattacharyya Distance (BD). The Quantum Jensen–Shannon divergence (QJSD) is used in pattern recognition [23], chemical physics [24], and other applications [25,26]. Additionally, the Kullback–Leibler Divergence proved its efficiency in different domains, in Fault Tolerant Fusion (FTF) approaches with FDI method for multi-robots localization in [27]. A general survey for the various informational divergences and measures used in the literature can be found in [28].

In the work that is cited above, in order to design residuals, the hypothesis, through the chosen information metric, is that the evolution model is always considered as a reference with respect to the observation model, despite the relevance of the cases where the observations are very reliable. Besides, in cases where the uncertainty may be high on both models, this assumption may lead to problems of missed detection in cases of possible faulty cases.

In this work, we propose a new way for generating these residuals by weighting, with a suitable way, the two covariance matrices (evolution model and observations) using α as a weight in the α-RD. Through the variation of α∈[0,∞], the α-RD generalizes a large number of possible informational measures. Corresponding residuals are then created based on proposed models for the fault-free cases and the faulty cases. After residual creation, an adaptive thresholding method that is based on α-Rényi criterion (α-Rc) is proposed in order to make decisions. In the case of error detection, an isolation algorithm based on Unknown Input Observers (UIO) is used to remove the error from the correction step of the chosen estimator.

The main contributions of this paper are: the development of a tightly coupling multi-sensor integration, the guarantee of the availability and the integrity thanks to an adaptive diagnostic layer able to detect both proprioceptive and exteroceptive sensors faults, the use of an advanced information metric, namely α-Rényi divergence, to design an adaptive and optimised thresholding strategy, and the validation of the approach with real experimental data.

This paper is organized, as follows: Section 2 presents the problem statement and introduces the Key Performance Indicators (KPIs). Section 3 presents the fusion architecture using Nonlinear Information Filter (NIF). Section 4 introduces the α-RD for fault detection and isolation, and introduces the balanced α as a proposed solution. Adaptive thresholding using the entropy based criterion is detailed in Section 5. The results with data from real experiment are shown and discussed in Section 6, followed by a conclusion in Section 7.

## 2. Problem Statement

The adoption of a technological solution as a means of localization of an Intelligent Transport System requires the validation of the usual key performance indicators. These are mainly accuracy, availability, continuity, and safety. Therefore, a sensor that is highly sensitive to the context in which it operates must be monitored with the appropriate diagnostic algorithm that can be adapted to the surrounding environment. Indeed, when one thinks not only of the vehicle, but also of the environment in which it evolves, it becomes obvious that the expected operational needs of the positioning system are also variable over time. Where, for example, the requirements in terms of expected precision or continuity are not the same in open sky as in urban canyon. Adding also the effect of relation between some of these KPIs, which can be sometimes contradictory. Taking for example, the availability and the safety which are two antagonism terms. So, one can see that achieving one KPI may affect the other(s). This link between the KPIs creates many important challenges that face the design of a robust, safe and available system. In this section, we highlight this relation by answering to the main operational challenges for any proposed estimation approach, then we deliver the proposed approach in details.

### 2.1. Diagnostic as a Guarantee of Safety

Safety requirements are expressed in terms of Tolerable Hazardous Rate (THR), which represents a maximum limit for iteration of system failures.

A very conservative diagnostic policy is required to achieve the objective. At the slightest suspicion of error, this policy will prefer to render the localization function unavailable. Here, we can see here the negative impact that this type of overly conservative policy can have on the availability and continuity of system. Having an adaptive diagnosis makes it possible to envisage relaxing the constraints of operational safety according to the context, all ensuring that the THR requested is not exceeded to improve availability.

In other words, a standard diagnostic allows for pursuing a single objective (i.e., the probability of false alarm or the probability of missed detection). Where, an adaptive diagnostic adds a degree of freedom allowing, depending on the change of environment and/or KPIs, reaching a higher level of sensitivity to the various faults, and to compromise between these two objectives.

### 2.2. Fault Tolerance as an Availability Booster

As we have seen, a conservative diagnostic can affect the localization function availability. Hence, in order to mitigate this problem and achieve continuous and safe positioning solutions, a fault tolerant layer must monitor the health of the sensor measurements and it analyzes the current situation to detect potentially dangerous and incipient or sudden changing situations, in order to provide an appropriate system behavior estimation to ensure a desired level of safety and maximum availability. In the literature, well known FDI algorithms that supervise and tolerate the fault are based on Solutions Separation(SS) [29]. These architectures are seemingly complex and very demanding in terms of computing resources, but they have the advantage of detecting a fault, only if it has an impact on the estimated position.

### 2.3. Proposed Approach Block Diagram

**Figure 1:**
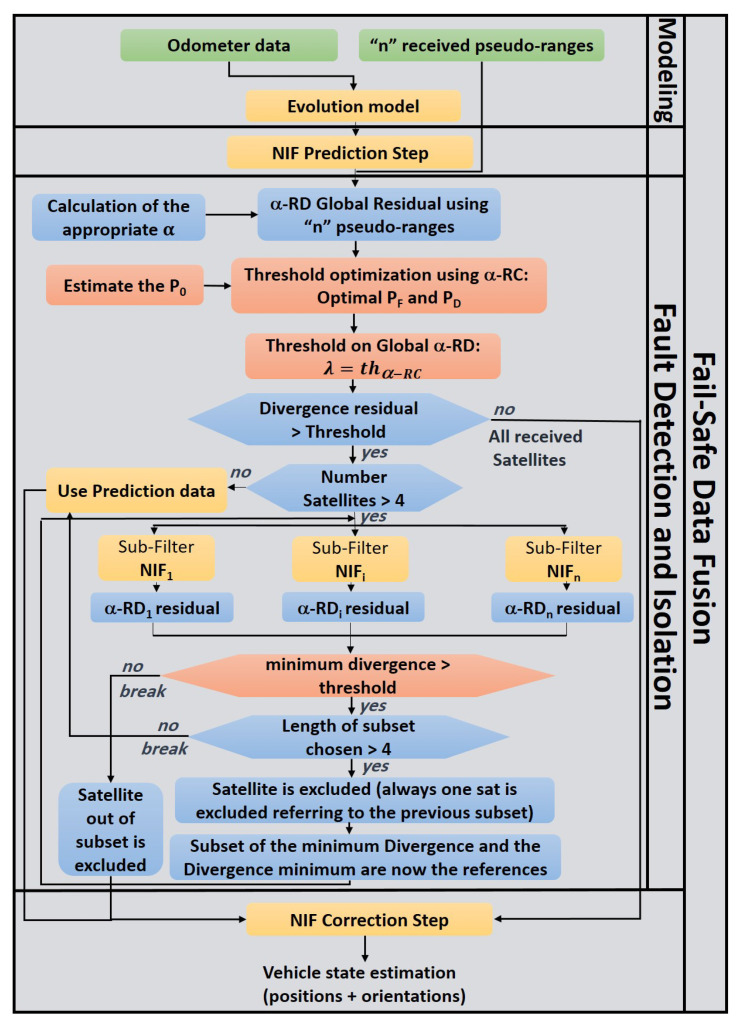
Detailed diagram for the proposed approach.

After presenting the main three challenges, we detail the proposed approach in its different levels. Figure 1 illustrates the proposed fault tolerant multi-sensor fusion approach. The algorithm is applied by fusing GNSS observations (pseudo-ranges) and odometer data using a Nonlinear Information Filter (NIF) as the main estimator.

After specifying the value of α thanks to a predefined objective function, all of the received observations are involved in the calculation of the global α-RD. The global residual is then tested with the threshold provided by the α-Rc. The α-Rc is used to provide the optimal missed detection probability ($P_{md}$) and detection probability ($P_{D}$) for each case. If the global residual is below the specified threshold, so the use of all observations in the correction step of the chosen NIF is safe for estimating the position. Where in the opposite case, the isolation step is activated to remove the erroneous measurements. The isolation method is a hierarchical algorithm that is based on the UIO, where, at each level, sets of the chosen IF sub-filters are created using the number of observations available at the previous level (n−1). At each level, local α-RD residuals are calculated for each sub-filter, and one erroneous satellite measurement is removed from the next level. This process continues until all of the erroneous measurements are removed and the final subset of observations has an α-RD below the threshold. Finally, the final safe subset is used in the correction step of the global NIF and the estimated position is calculated.

Noting that, during the whole process, the number of satellites is taken into consideration. It should not be less than four satellites at instant *k* in order to deliver a position [30].

## 3. Nonlinear Information Filter

The information form of the EKF, called NIF, is used in order to estimate the position of the vehicle [21]. As the EKF, the NIF consists of two main steps, called the prediction and correction steps, using the covariance matrix as information matrix and the state vector as information vector.

Consider the following non-linear system:

(1) $$X_{k}=f\left(X_{k-1},u_{k}\right)+w_{k}$$

where, $X_{k}$ is the state vector, f(.) is the non-linear function, $u_{k}$ is the input vector, and $w_{k}$$∼N(0,\;Q_{k})$ is the model noise that is considered as white Gaussian noise of zero mean value and covariance matrix $Q_{k}$.

The non-linear observation model has the following form:

(2) $$Z_{k}=h\left(X_{k},\epsilon_{k}\right)$$

where, $Z_{k}$ is the observations vector and $\epsilon_{k}$ is the observation noise vector that is considered as white Gaussian noise of zero mean value and covariance matrix $R_{k}=E\left[\epsilon_{k}\epsilon_{k}^{T}\right]$.

The first step of the NIF is called the prediction step, and it uses the following information matrix and information vector to predict the position:

(3) $$Y_{k/k-1}={\left[F_{k}P_{k-1/k-1}F_{k}^{T}+B_{k}Q_{k}^{u}B_{k}^{T}+Q_{k}\right]}^{-1}$$

(4) $$y_{k/k-1}=Y_{k/k-1}X_{k/k-1}$$

with: 

$Q_{k}^{u}$ the input vector variance-covariance matrix,

$F_{k}$ the Jacobian matrix of *f*: $F_{k}=\frac{\partial f}{\partial X}{|}_{X=X_{k-1/k-1}}$,

$B_{k}$ the Jacobian matrix calculated as: $B_{k}=\frac{\partial f}{\partial u}{|}_{u=u_{k}}$.

These equations are written in the second step, called the correction step, as follows:

(5) $$Y_{k/k}=Y_{k/k-1}+\sum_{i=1}^{N}gI_{i}\left(k\right)$$

(6) $$y_{k/k}=y_{k/k-1}+\sum_{i=1}^{N}pI_{i}\left(k\right)$$

where $gI_{i}\left(k\right)$ and $pI_{i}\left(k\right)$ are seen as information contribution calculated as:

(7) $$gI_{i}\left(k\right)=H_{i,k}^{T}R_{i}^{-1}\left(k\right)H_{i,k}$$

(8) $$pI_{i}\left(k\right)=H_{i,k}^{T}R_{i}^{-1}\left(k\right)\left[\left(Z_{i,k}-{\hat{Z}}_{i,k}\right)+H_{i,k}X_{k/k-1}\right]$$

Additionally, *N* is the number of observations at instant *k*.

## 4. Adaptive Diagnostic Layer Based on α-Rényi Divergence

One of the problems faced in diagnosis is the use of one chosen model when the system evolves in a dynamic environment and in different conditions. An effective diagnostic layer has to be presented qfter a robust sensors fusion. In this work, the α-RD for residual design is proposed, then, the features and advantages for this divergence are presented in detail.

The α-RD choice is based on the wide range of divergence measures offered, by changing the value of α [31], which makes the divergence more flexible with the type of application.

### 4.1. α-Rényi Divergence as a Parametric Residual

For two probability distributions *P* and *Q*, the α-RD between *P* and *Q* is non-decreasing as a unction of its order α, and it is continuous on the set of α for which it is finite. It can be written as:

(9) $$RD_{\alpha }\left(P||Q\right)=\frac{1}{\alpha -1}ln\int P^{\alpha }\left(x\right)Q^{1-\alpha }\left(x\right)d\left(x\right)$$

where α∈R−{1} [32].

The generation of residuals through α-RD makes the residual more flexible and adaptable with the environment, the dynamic, and the kind of errors. Hence, to calculate the divergence between the two probability distribution functions (*pdfs*) g(k/k−1)$∼N(X_{k/k-1},\Sigma_{k/k-1})$ and g(k/k)$∼N(X_{k/k},\Sigma_{k/k})$, representing the two covariance matrices of the prediction and the correction steps that are provided by the chosen NIF estimator, the α-RD can be written as the following equation [33]:

(10) $$\begin{array}{cc} \; & RD_{\alpha }\left(g\left(k/k-1\right)||g\left(k/k\right)\right)=\\ \; & \frac{\alpha }{2}{\left(X_{k/k-1}-X_{k/k}\right)}^{T}{\left(\Sigma_{\alpha }\right)}^{-1}\left(X_{k/k-1}-X_{k/k}\right)-\frac{1}{2(\alpha -1)}\log\frac{|\Sigma_{\alpha }|}{|\Sigma_{k/k-1}{|}^{1-\alpha }{\left|\Sigma_{k/k}\right|}^{\alpha }} \end{array}$$

Taking into consideration that $\Sigma_{\alpha }=\alpha \Sigma_{k/k}+\left(1-\alpha \right)\Sigma_{k/k-1}$, $\Sigma_{k/k-1}=\frac{1}{Y_{k/k-1}}$, and $\Sigma_{k/k}=\frac{1}{Y_{k/k}}$.

Hence, based on Appendix A, the Equation (10) can be written in the following form:

(11) $$\begin{array}{cc} \; & RD_{\alpha }\left(g\left(k/k-1\right)||g\left(k/k\right)\right)=\\ \; & \frac{\alpha }{2}{\left(X_{k/k-1}-X_{k/k}\right)}^{T}\left(\frac{Y_{k/k-1}Y_{k/k}}{\alpha Y_{k/k-1}+\left(1-\alpha \right)Y_{k/k}}\right)\left(X_{k/k-1}-X_{k/k}\right)+\\ \; & \frac{1}{2(\alpha -1)}\log\left|\frac{Y_{k/k-1}Y_{k/k}}{\alpha Y_{k/k-1}+\left(1-\alpha \right)Y_{k/k}}\right|+\frac{1}{2(\alpha -1)}\log\frac{|Y_{k/k-1}{|}^{\alpha -1}}{|Y_{k/k}{|}^{\alpha }} \end{array}$$

This residual consists of three kind of tests that deal with the two *pdfs* in different ways:

1. The first test: ${\left(X_{k/k-1}-X_{k/k}\right)}^{T}\left(\frac{Y_{k/k-1}Y_{k/k}}{\alpha Y_{k/k-1}+\left(1-\alpha \right)Y_{k/k}}\right)\left(X_{k/k-1}-X_{k/k}\right)$
  represented by the weighted Mahalanobis distance, is to measure the distance between the means, while taking the α value into consideration, which weights distance through its impact on the covariance matrices,
2. The second test: $\log|\frac{Y_{k/k-1}Y_{k/k}}{\alpha Y_{k/k-1}+\left(1-\alpha \right)Y_{k/k}}|$, can be compared to the weighted Bregman, while taking the weight of α for each covariance matrice into account,
3. The third test: $\log\frac{|Y_{k/k-1}{|}^{\alpha -1}}{|Y_{k/k}{|}^{\alpha }}$, represented by the weighted MI, where, in this test, the two *pdfs* are compared based on their weighting value related to the value of α.

These tests creating the generalized α-RD residual, show a weighty flexibility related to the value of α.

### 4.2. Establish a Residuals Parameterization Policy Based on Operational Requirements and Changes in Navigation Context

Taking into account the different purposes for the use of an adaptive diagnosis explained in the previous sections, the adaptive diagnosis is proposed in this section based on multiple reasons. First, we are dealing with two models, the evolution model and the observation model. The limitations that restrain the odometer sensor, as well as the limitations of GNSS, due to many different causes that are listed in [34], lead us to completely rely on neither of the two models.

The infinity of particular cases that are generalized by α-RD correspond to a particular appreciation of the trust between the evolution model and the observations. In the case of the Kullback Leibler (α→1), the residuals are generally designed to only detect observation faults and all of the confidence is given to the evolution model. To be effective, the model must be based on mathematical knowledge of the system. For this knowledge to be precise, the model must be complex and hard to establish. Otherwise, if the model is not very accurate, the uncertainty fixed around this model must be large. This reduces the possibility of fault detection.In the case of Bhattacharya divergence, the appreciation gauge is placed in the middle and neither of the two models is a priori privileged. However, there remains an imbalance due to the original covariance of each of the models. For the choice of the α value at each instant *k*, and knowing that there is no reliable reference between the two models, we propose a solution to weight the two models equally through α balance. The following equation takes into consideration the covariance matrix $\Sigma_{k/k-1}$ of the prediction step representing the evolution model, and the covariance matrix $\Sigma_{k/k}$ of the correction step representing the observation model, then equally weight the matrices.

(12) $$\alpha |\Sigma_{k/k-1}\left|=\left(1-\alpha \right)\right|\Sigma_{k/k}|$$

this equation leads to the weighted value of α:

(13) $$\alpha =\frac{|\Sigma_{k/k}|}{|\Sigma_{k/k-1}\left|+\right|\Sigma_{k/k}|}$$

which is written in informational form as:

(14) $$\alpha =\frac{|Y_{k/k-1}|}{|Y_{k/k}\left|+\right|Y_{k/k-1}|}$$

One can notice that, regardless of their original weights, using the equality in Equation (14), the weight is equally distributed between the prediction and correction *pdfs*. The adaptive α avoids the problem of model’s reference faced along the trajectory by proposing a new residual at each instant *k*.

### 4.3. An Infinity of Residuals Implies an Infinity of Statistical Characterization … How to Solve?

**Figure 2:**
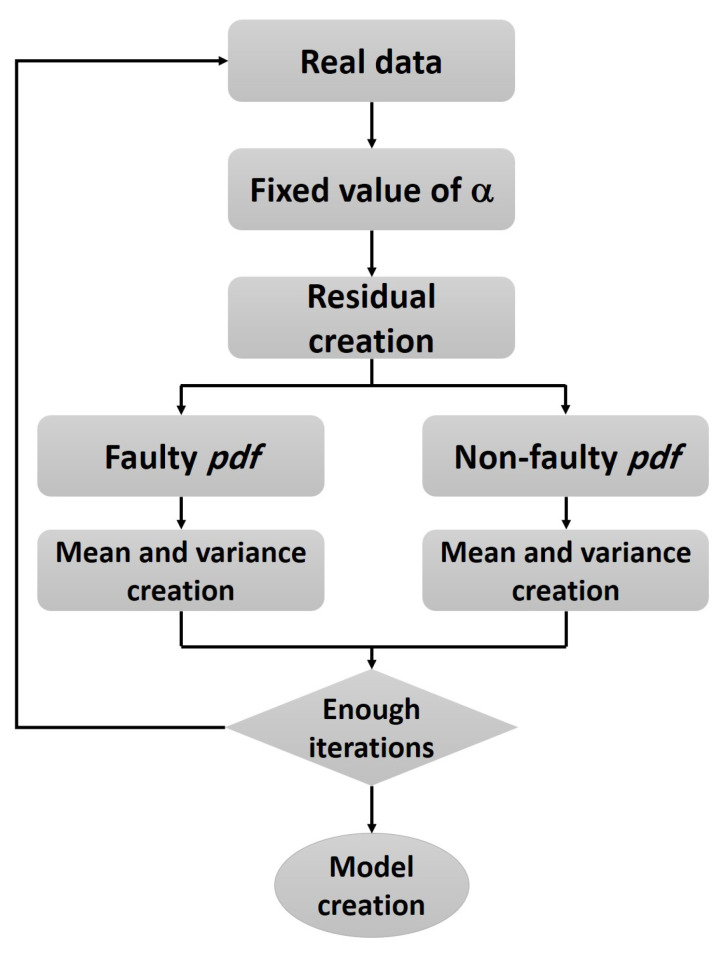
Diagram to create two probability distributions.

The policy that is chosen in treating the adaptive diagnosis leads to an infinity of residuals, which implies an infinity of statistical characterization. In fact, creating the two *pdfs* for an infinite values of α is unthinkable. Hence, to avoid such problems, one of the solutions is by creating mathematical models that are able to estimate the two *pdfs*. The diagram presented in Figure 2 shows the procedure that is used to create these models.

**Figure 3:**
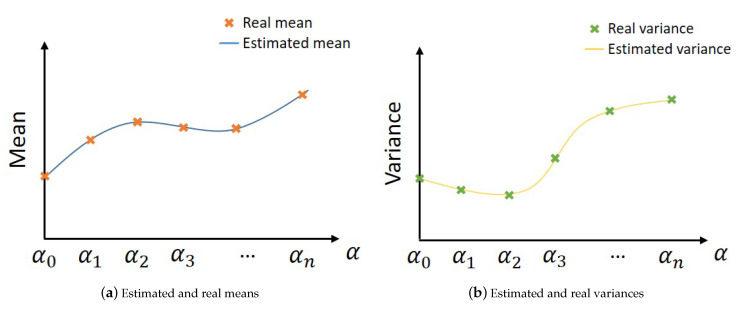
Extracting estimated means and variances from real values.

Using real sensors data, for many values of α, residuals are calculated and the two *pdfs* for faulty and non-faulty cases are created through a non-supervised learning method for each value of α. Subsequently, the means and the variances are extracted and plotted with respect to α. Hence, a function that approximates these values is created. This function represents the mathematical model to estimate a new value of α (Figure 3).

The mean and variance models created take the following mathematical form:

1. For non-faulty cases:
  (15) $$\begin{array}{c} M_{nf}=\sum_{i=1,n}a_{i}\alpha^{i}\\ V_{nf}=\sum_{j=1,n}b_{j}\alpha^{j} \end{array}$$
2. For faulty cases:
  (16) $$\begin{array}{c} M_{f}=\sum_{k=1,n}c_{k}\alpha^{k}\\ V_{f}=\sum_{m=1,n}d_{m}\alpha^{m} \end{array}$$

Where *a*, *b*, *c*, and *d* are considered to be constant values. Using the above models, one is able to approximate the mean and variance for the new value of α in order to create the two *pdfs*.

## 5. α-Rényi Criterion Generalizing Threshold Optimization

### 5.1. α-Rc Design

Based on Equation (9), the informational gain that is associated to the decision $u_{j}$, represents the α-Rényi divergence between the *a priori* and *a posteriori* distributions. This gain can be written as:

(17) $$RD_{\alpha }(p\left(H/u_{j}\right)||p\left(H\right))=\frac{1}{\alpha -1}\log\sum_{i\in \{0,1\}}{\left(p\left(H_{i}/u_{j}\right)\right)}^{\alpha }{\left(p\left(H_{i}\right)\right)}^{1-\alpha }$$

Therefore, the α-Rényi criterion that is represented by the summation of the informational gain corresponding to decisions $u_{0}$ and $u_{1}$, is written as:

(18) $$Rc_{\alpha }=RD_{\alpha }(p\left(H/u_{0}\right)||p\left(H\right))+RD_{\alpha }(p\left(H/u_{1}\right)||p\left(H\right))$$

which leads to:

(19) $$Rc_{\alpha }=\frac{1}{\alpha -1}\sum_{i\in \{0,1\}}\log\sum_{j\in \{0,1\}}{\left(\frac{p(u_{j}/H_{i})}{p(u_{j})}\right)}^{\alpha }p\left(H_{i}\right)$$

Thus, the α-Rényi criterion is written as:

(20) $$\begin{array}{cc} \; & Rc_{\alpha }=\frac{1}{\alpha -1}\log P_{0}\left(1-P_{0}\right)\\ \; & +\frac{1}{\alpha -1}\log\left[{\left[P_{0}\gamma_{1}\gamma_{0}+\left(1-P_{0}\right)\beta_{1}\gamma_{0}\right]}^{\alpha }+{\left[P_{0}\gamma_{1}\gamma_{0}+\left(1-P_{0}\right)\gamma_{1}\beta_{0}\right]}^{\alpha }\right]\\ \; & +\frac{1}{\alpha -1}\log\left[{\left[P_{0}\beta_{1}\gamma_{0}+\left(1-P_{0}\right)\beta_{1}\beta_{0}\right]}^{\alpha }+{\left[P_{0}\gamma_{1}\beta_{0}+\left(1-P_{0}\right)\beta_{1}\beta_{0}\right]}^{\alpha }\right]\\ \; & -\frac{2\alpha }{\alpha -1}\log\left[P_{0}\gamma_{0}+\left(1-P_{0}\right)\beta_{0}\right]\\ \; & -\frac{2\alpha }{\alpha -1}\log\left[P_{0}\gamma_{1}+\left(1-P_{0}\right)\beta_{1}\right] \end{array}$$

where: $\gamma_{0}=1-P_{F}$, $\beta_{0}=1-P_{D}$, $\gamma_{1}=P_{F}$, $\beta_{1}=P_{D}$, $P_{F}$ is false alarm of the probability and $P_{0}$ is calculated using the following equation:

(21) $${\hat{P}}_{0}=1-\frac{\sum_{i=1}^{n}h^{i}}{n}$$

where $h^{i}=0$ if the decision is $H_{0}$ or $h^{i}=1$ if the decision is $H_{1}$. Accordingly, $\sum_{i=1}^{n}h^{i}$ represents the windows taken for *n* past hypotheses.

### 5.2. Variation of α-Rc

The variation of α-Rc is studied through the derivative of the α-Rc with respect to a given variable *v*. This derivative is calculated based on Equation (20), and it is written as:

(22) $$\begin{array}{cc} \; & \frac{\partial Rc_{\alpha }}{\partial v}=\frac{\alpha }{\alpha -1}[\\ \; & \frac{\sum_{i\in \{0,1\}}\gamma_{i}^{\alpha -1}{\left[P_{0}\gamma_{\bar{i}}+\left(1-P_{0}\right)\beta_{\bar{i}}\right]}^{\alpha -1}\left[\left(P_{0}\gamma_{0}-P_{0}\gamma_{1}+{\left(-1\right)}^{i+1}\left(1-P_{0}\right)\beta_{\bar{i}}\right)\frac{\partial \gamma_{1}}{\partial v}+{\left(-1\right)}^{i+1}\left(1-P_{0}\right)\gamma_{i}\frac{\partial \beta_{1}}{\partial v}\right]}{\sum_{i\in \{0,1\}}\gamma_{i}^{\alpha }{\left[P_{0}\gamma_{\bar{i}}+\left(1-P_{0}\right)\beta_{\bar{i}}\right]}^{\alpha }}\\ \; & +\frac{\sum_{i\in \{0,1\}}\beta_{i}^{\alpha -1}{\left[P_{0}\gamma_{\bar{i}}+\left(1-P_{0}\right)\beta_{\bar{i}}\right]}^{\alpha -1}\left[\left({\left(-1\right)}^{i+1}\gamma_{\bar{i}}+\left(1-P_{0}\right)\beta_{0}-\left(1-P_{0}\right)\beta_{1}\right)\frac{\partial \beta_{1}}{\partial v}+{\left(-1\right)}^{\bar{i}+1}\beta_{i}\frac{\partial \gamma_{1}}{\partial v}\right]}{\sum_{i\in \{0,1\}}\beta_{i}^{\alpha }{\left[P_{0}\gamma_{\bar{i}}+\left(1-P_{0}\right)\beta_{\bar{i}}\right]}^{\alpha }}\\ \; & -2\sum_{i\in \{0,1\}}\frac{P_{0}\frac{\partial \gamma_{i}}{\partial v}+\left(1-P_{0}\right)\frac{\partial \beta_{i}}{\partial v}}{P_{0}\gamma_{i}+\left(1-P_{0}\right)\beta_{i}}] \end{array}$$

1. for $P_{F}=constant$, the derivative of α-Rc with respect to $P_{D}$ is written as:
  (23) $$\begin{array}{cc} \; & \frac{\partial Rc_{\alpha }}{\partial P_{D}}=\frac{\alpha }{\alpha -1}[\left(1-P_{0}\right)\frac{\sum_{i\in \{0,1\}}{\left(-1\right)}^{i+1}\gamma_{\bar{i}}^{\alpha }{\left[P_{0}\gamma_{i}+\left(1-P_{0}\right)\beta_{i}\right]}^{\alpha -1}}{\sum_{i\in \{0,1\}}\gamma_{i}^{\alpha }{\left[P_{0}\gamma_{\bar{i}}+\left(1-P_{0}\right)\beta_{\bar{i}}\right]}^{\alpha }}\\ \; & +\frac{\sum_{i\in \{0,1\}}\beta_{i}^{\alpha -1}{\left[P_{0}\gamma_{\bar{i}}+\left(1-P_{0}\right)\beta_{\bar{i}}\right]}^{\alpha -1}\left[{\left(-1\right)}^{i+1}P_{0}\gamma_{\bar{i}}+\left(1-P_{0}\right)\left(1-2\beta_{1}\right)\right]}{\sum_{i\in \{0,1\}}\beta_{i}^{\alpha }\left[{\left[P_{0}\gamma_{\bar{i}}+\left(1-P_{0}\right)\beta_{\bar{i}}\right]}^{\alpha }\right]}\\ \; & -2\sum_{i\in \{0,1\}}\frac{{\left(-1\right)}^{i+1}\left(1-P_{0}\right)}{P_{0}\gamma_{i}+\left(1-P_{0}\right)\beta_{i}}] \end{array}$$
  Based on Equation (23):
  $\frac{\partial Rc_{\alpha }}{\partial P_{D}}=0$ if $P_{D}=P_{F}$,
  $\frac{\partial Rc_{\alpha }}{\partial P_{D}}>0$ if $P_{D}>P_{F}$,
  $\frac{\partial Rc_{\alpha }}{\partial P_{D}}<0$ if $P_{D}<P_{F}$.
  We conclude that α-Rc is a decreasing function on $[0,P_{D}[$ and an increasing function on $]P_{D},1]$ with a minimum point reached at $P_{D}=P_{F}$. Hence, by maximizing the α-Rc, the $P_{D}$ is maximized when $P_{F}$ is fixed.
2. for $P_{D}=constant$, the derivative of α-Rc with respect to $P_{F}$ is written as:
  (24) $$\begin{array}{cc} \; & \frac{\partial Rc_{\alpha }}{\partial P_{F}}=\frac{\alpha }{\alpha -1}[P_{0}\frac{\sum_{i\in \{0,1\}}{\left(-1\right)}^{i+1}\beta_{\bar{i}}^{\alpha }{\left[P_{0}\gamma_{i}+\left(1-P_{0}\right)\beta_{i}\right]}^{\alpha -1}}{\sum_{i\in \{0,1\}}{\left(-1\right)}^{i+1}\beta_{i}^{\alpha }{\left[P_{0}\gamma_{\bar{i}}+\left(1-P_{0}\right)\beta_{\bar{i}}\right]}^{\alpha }}\\ \; & \frac{\sum_{i\in \{0,1\}}\gamma_{i}^{\alpha -1}{\left[P_{0}\gamma_{\bar{i}}+\left(1-P_{0}\right)\beta_{\bar{i}}\right]}^{\alpha -1}\left[P_{0}+{\left(-1\right)}^{i+1}\left(1-P_{0}\right)\beta_{\bar{i}}-2P_{0}\gamma_{1}\right]}{\sum_{i\in \{0,1\}}\gamma_{i}^{\alpha }{\left[P_{0}\gamma_{\bar{i}}+\left(1-P_{0}\right)\beta_{\bar{i}}\right]}^{\alpha }}\\ \; & -2\sum_{i\in \{0,1\}}\frac{{\left(-1\right)}^{i+1}P_{0}}{P_{0}\gamma_{i}+\left(1-P_{0}\right)\beta_{i}}] \end{array}$$
  Based on Equation (24):
  $\frac{\partial Rc_{\alpha }}{\partial P_{F}}=0$ if $P_{F}=P_{D}$,
  $\frac{\partial Rc_{\alpha }}{\partial P_{F}}>0$ if $P_{F}>P_{D}$,
  $\frac{\partial Rc_{\alpha }}{\partial P_{F}}<0$ if $P_{F}<P_{D}$.
  We conclude that α-Rc is a decreasing function on $[0,P_{F}[$ and an increasing function on $]P_{F},1]$ with a minimum point reached at $P_{F}=P_{D}$. Accordingly, by maximizing the α-Rc, the $P_{F}$ is minimized when $P_{D}$ is fixed.
  As conclusion, in order to minimize the false alarm probability and maximize the detection probability, it is equivalent to maximizing the α-Rc.

### 5.3. Threshold Optimization Algorithm

The threshold corresponding to these conditions is found by the maximization of α-Rc, as we are interested in maximizing the detection probability and minimizing the false alarm probability.

Starting from the Bayes rule:

(25) $$\frac{p\left(x/H_{1}\right)p\left(H_{1}\right)}{p(x)}\overset{H_{1}}{\underset{H_{0}}{≷}}\frac{p\left(x/H_{0}\right)p\left(H_{0}\right)}{p(x)}$$

which leads to the likelihood ratio test that is written as:

(26) $$\Lambda =\frac{p(x/H_{1})}{p(x/H_{0})}\overset{H_{1}}{\underset{H_{0}}{≷}}\frac{p(H_{0})}{p(H_{1})}$$

and by setting the derivative of α-Rc in Equation (22) to zero ($\frac{\partial Rc_{\alpha }}{\partial v}{|}_{v=th}=0$), the likelihood ratio, linked to the threshold $\frac{\partial P_{D}}{\partial P_{F}}=\Lambda$, which maximizes the α-Rc, is written as:

(27) $$\Lambda \overset{H_{1}}{\underset{H_{0}}{≷}}\frac{a\left(A_{01}+A_{10}\right)+b\left(B_{01}+B_{10}\right)+\frac{PM}{V})}{C_{01}\left(A_{01}-A_{10}\right)-C_{10}\left(A_{01}+A_{10}\right)+d\left(B_{01}+B_{10}\right)+\frac{PN}{V})}$$

where:

(28) $$\begin{array}{cc} l_{ij} & =P_{0}\beta_{j}\gamma_{i}+\left(1-P_{0}\right)\beta_{j}\beta_{i}\\ n_{ij} & =P_{0}\gamma_{i}\gamma_{j}+\left(1-P_{0}\right)\beta_{j}\gamma_{i}\\ A_{ij} & =l_{ij}^{\alpha },\;B_{ij}=n_{ij}^{\alpha },\;C_{ij}=n_{ij}^{\alpha -1}\left(1-P_{0}\right)\gamma_{0}\\ a & =\left[-P_{0}\left(\gamma_{0}-\gamma_{1}\right)+\left(1-P_{0}\right)\beta_{1}\right]n_{ij}^{\alpha -1}+\left[-P_{0}\left(\gamma_{0}-\gamma_{1}\right)-\left(1-P_{0}\right)\beta_{0}\right]n_{ji}^{\alpha -1}\\ b & =l_{ij}^{\alpha -1}P_{0}\beta_{1}-l_{ji}^{\alpha -1}P_{0}\beta_{0}\\ d & =P_{0}\gamma_{0}-\left(1-P_{0}\right)\left(\beta_{0}+\beta_{1}\right)l_{ij}^{\alpha -1}-P_{0}\gamma_{1}+\left(1-P_{0}\right)\left(\beta_{0}-\beta_{1}\right)l_{ji}^{\alpha -1}\\ P & =\left(n_{ij}^{\alpha }+n_{ji}^{\alpha }\right)\left(l_{ij}^{\alpha }+l_{ji}^{\alpha }\right)\\ M & =2P_{0}^{2}\left(\gamma_{0}-\gamma_{1}\right)+2P_{0}\left(1-P_{0}\right)\left(\beta_{0}-\beta_{1}\right)\\ N & =2\left(1-P_{0}\right)\left(\gamma_{1}-\gamma_{0}\right)+2{\left(1-P_{0}\right)}^{2}\left(\beta_{1}-\beta_{0}\right)\\ V & =P_{0}^{2}\gamma_{0}\gamma_{1}+\left(1-P_{0}\right)P_{0}\gamma_{0}\beta_{1}+\left(1-P_{0}\right)P_{0}\beta_{0}\gamma_{1}+{\left(1-P_{0}\right)}^{2}\beta_{0}\beta_{1} \end{array}$$

with:

$$\begin{array}{c} i=\bar{j}\in \left\{0,1\right\} \end{array}$$

The threshold optimization algorithm is given, as follows (Algorithm 1):
**Algorithm 1:** Threshold optimization based on α-Rc
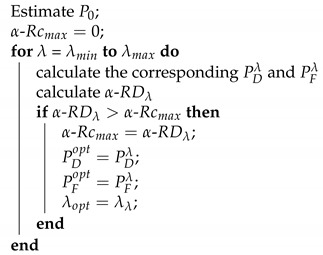


## 6. Experimental Results

With the use of a robust filter for sensors fusion that can ensure the availability of the system and, in most of cases, the accuracy as well, the need of the safety attribute is necessary. Hence, a diagnostic layer that is based on α-RD to deliver the safety and avoid accuracy problems caused by feared events is proposed. In order to validate our proposed approach, experimental results using an experimental vehicle, the equipped autonomous vehicle of the CRIStAL Lab, are shown in the following section.

Thus, two scenarios are presented based on the proposed α-RD for residuals generation. The first scenario deals with fixed α value in the whole trajectory, where the second one deals with balanced α. The results are shown and a result comparison is done in the end of the two presented scenarios.

**Figure 4:**
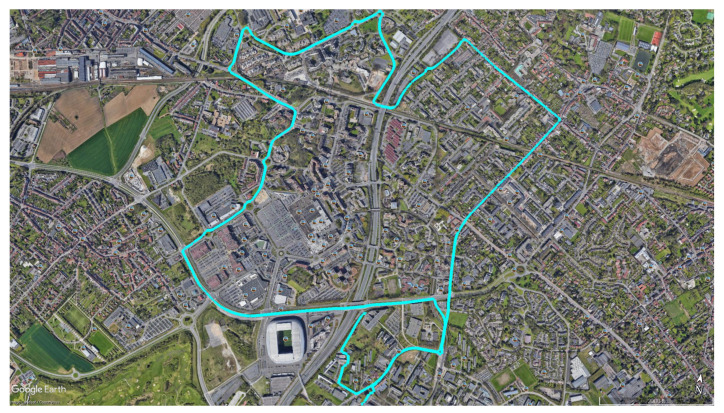
Trajectory C3 reference.

Noting that, in both scenarios, the same trajectory C3 seen in Figure 4 and detailed in the Table 1, is compared with the same conditions and the same collected data.

### 6.1. Results without FDI Approach

**Figure 5:**
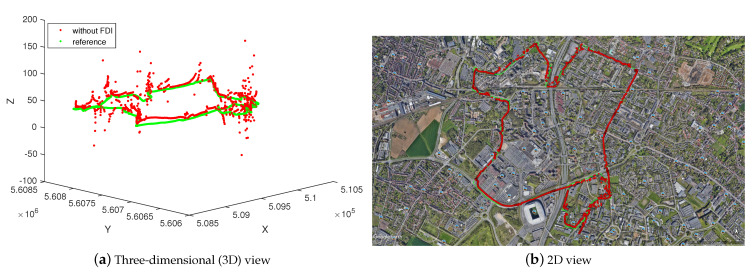
Positioning estimation without Fault Detection and Isolation (FDI) algorithm vs reference in three/two-dimensional (3D/2D) views.

The following results in Figure 5 illustrate the positioning estimation without any FDI algorithm on the trajectory C3 (Figure 4). In both views, one can see the level of error presenting especially in the z-axis.

**Figure 6:**
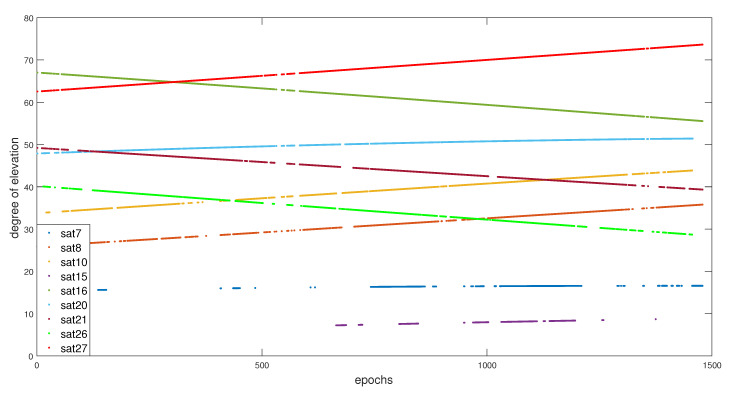
Elevation of each satellites during the whole trajectory.

These errors are due to either the multipaths that are caused by signals reflection, Non Line-Of-Sight (NLOS), or related to the low elevation of the satellites that are available at the instant *k*. The elevation of all satellites available in this trajectory can be seen in Figure 6.

**Figure 7:**
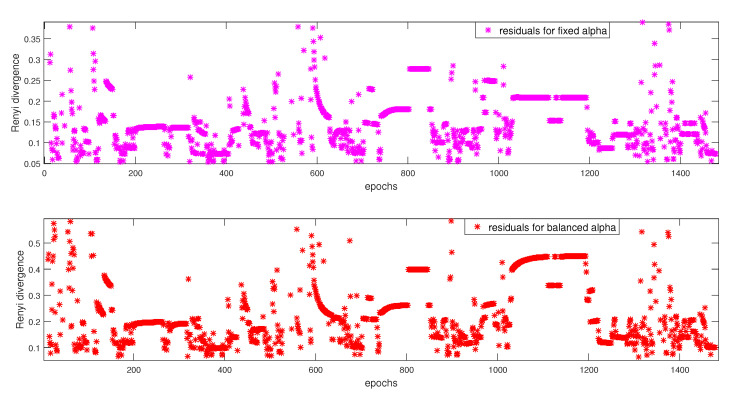
α-Rényi Residuals without FDI.

We provide the results with the FDI proposed algorithm using both the fixed and balanced α strategy in order to detect and isolate these errors. However, before going to the next section, Figure 7 illustrates the global α-Rényi residuals for the fixed and balanced α. These residuals that are calculated without the FDI approach show disarrayed alignments, which indicate the presence of faults.

### 6.2. Results with FDI Approach

A good residual design reduces the loss of some fault information, as mentioned in Section 4. From this point, we deliver the results for two different scenarios. In the first scenario, residuals are generated through fixed α with α value equal to 0.5 which is related to the Bhattacharyya divergence [35]. The second implements the α balance strategy.

#### 6.2.1. Residual Design Using Fixed α

**Figure 8:**
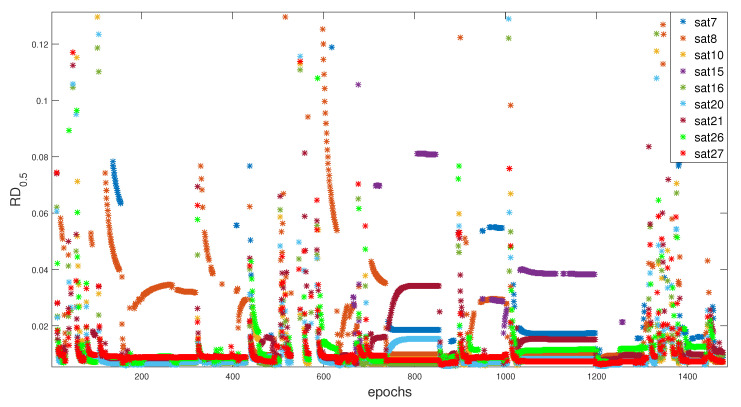
Partials $RD_{0.5}$ observation for identification.

The residuals that are generated by the fixed RD for each satellites during the whole trajectory can be seen in Figure 8.

**Figure 9:**
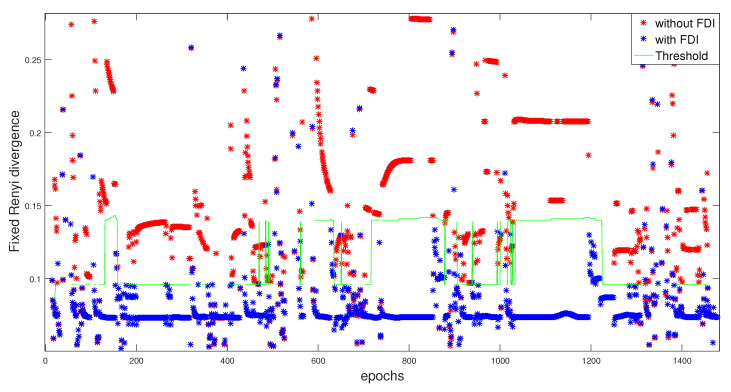
$RD_{0.5}$ divergence without/with FDI with adaptive threshold.

The behavior of each satellite is monitored at each epoch during the whole trajectory. The detection of any erroneous behavior is carried out while using the Rényi criterion (for α=0.5) by optimizing the threshold value. This threshold is illustrated with the global residuals, without and with the FDI approach, in the Figure 9.

**Figure 10:**
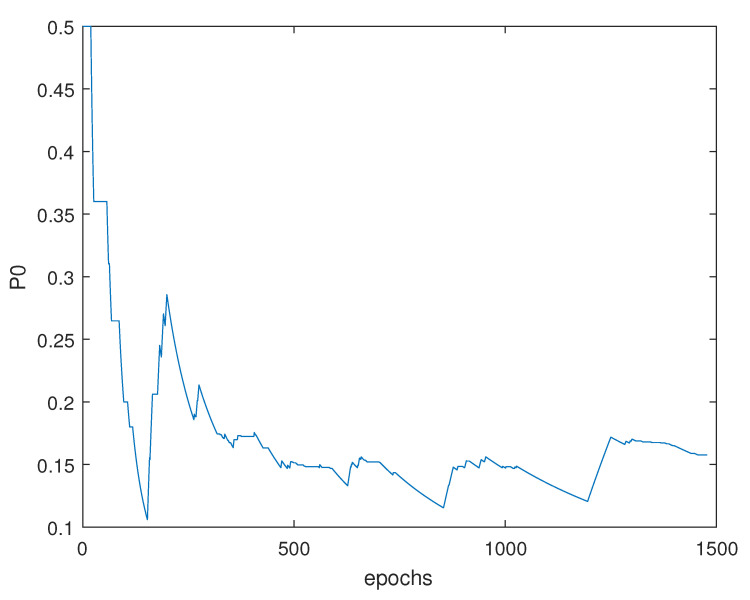
P0 during the whole trajectory.

As we can see, the threshold is adaptive to the residuals, which means that it is calculated based on a variable that is related to the decisions. This variable is $P_{0}$, which takes into consideration the last 10 decisions during the past epochs, and can be calculated using Equation (21). The $P_{0}$ represents the threshold in Figure 9 that can be seen in Figure 10.

The behavior of $P_{0}$ indicates the low probability to be in a case where no fault will be detected. Where, the value increases as much as an isolation step is conducted.

#### 6.2.2. Residual Design Using α Balanced

**Figure 11:**
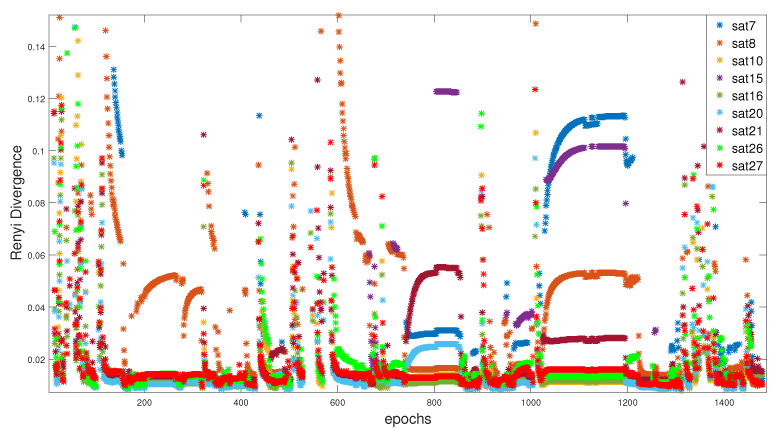
Partials Rényi observation for identification.

The same structure is followed in this section using the α balanced. Where, the α-Rényi residuals for each satellite are presented in the Figure 11.

**Figure 12:**
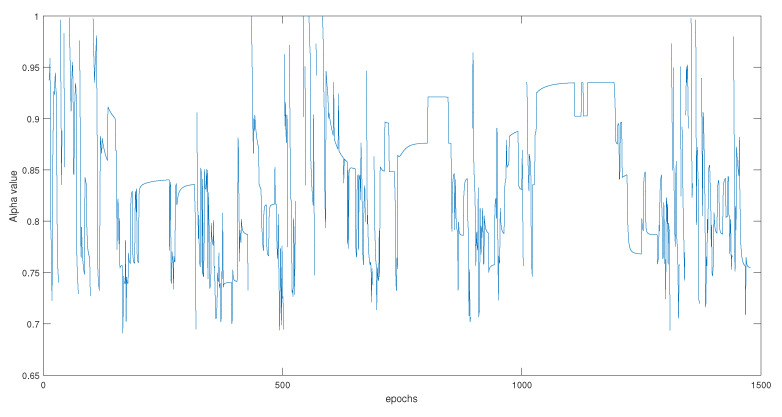
Weighted Alpha balance.

Many of the available satellites carry an erroneous information that should be isolated from the final estimation, like satellites 7 (dark blue), 15 (mauve), and 8 (orange). Unlike $RD_{0.5}$, these residuals are generated by equally ’reweighting’ the prediction and correction covariance matrices. The value of α calculated to weight in a manner to equally take into consideration the two uncertainties, while using Equation (14), can be seen in Figure 12.

Subsequently, the detection is occurred using an adaptive threshold that is optimized by the α-Rc, where each residual is judged with its corresponding criterion. Hence, referring to the problem that is discussed in Section 4.3, where an infinity of residuals implies an infinity of statistical characterization, we used the proposed models to estimate the mean and variance for the corresponding value of α.

**Figure 13:**
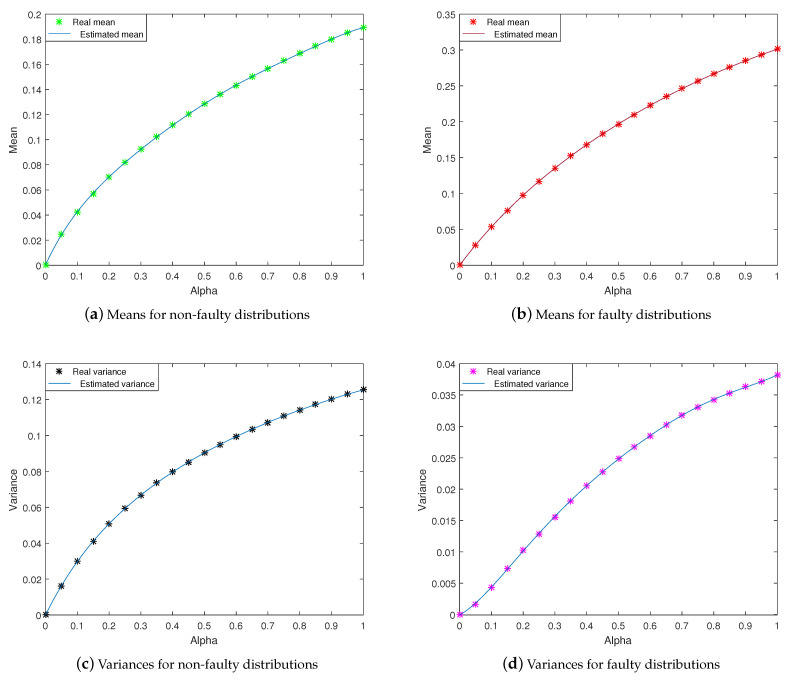
Estimated and real means & variances for faulty and non-faulty distributions.

In our application, the models for estimating the means and the variances of faulty and non-faulty distributions are created by fitting the real samples (Figure 13).

The fitting functions for the real means and variances lead to the following mathematical models for estimating the two distributions of other value of α:

- For non-faulty cases:
  (29) $$\begin{array}{cc} M_{nf}= & -1.2482\alpha^{6}+4.2123\alpha^{5}-5.6404\alpha^{4}\\ \; & +3.8677\alpha^{3}-1.5455\alpha^{2}+0.54279\alpha +0.00039907 \end{array}$$
  (30) $$\begin{array}{cc} V_{nf}= & -0.24207\alpha^{6}+0.91051\alpha^{5}-1.4299\alpha^{4}\\ \; & +1.2507\alpha^{3}-0.72262\alpha^{2}+0.3587*\alpha +6.3904e^{-06} \end{array}$$
- For faulty cases:
  (31) $$\begin{array}{cc} M_{f}= & 0.11628\alpha^{5}-0.4186\alpha^{4}+0.62826\alpha^{3}\\ \; & -0.61309\alpha^{2}+0.58827\alpha +9.2291e^{-05} \end{array}$$
  (32) $$\begin{array}{cc} V_{f}= & 0.29775\alpha^{6}-0.9601\alpha^{5}+1.2201\alpha^{4}\\ \; & -0.77397\alpha^{3}+0.22616\alpha^{2}+0.028363\alpha -7.1952e^{-05} \end{array}$$

These models are created by fitting the real values of means and variances, taking twenty values of α between [0,1]. Where, the real means and variances are extracted using real experimental *pdfs*.

**Figure 14:**
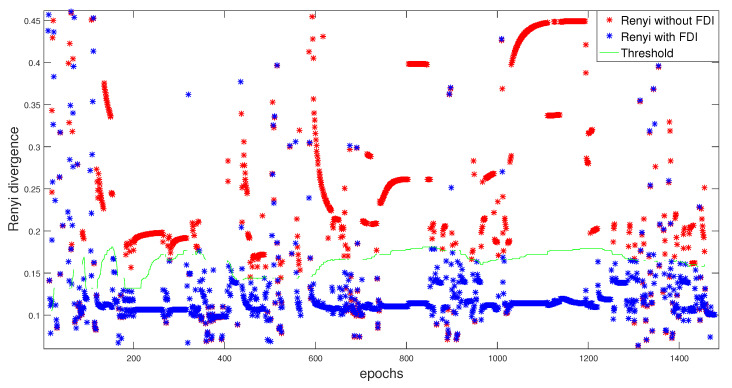
Rényi divergence without/with FDI with adaptive threshold.

After generating the statistical characterization that is used for α-Rc optimization, the adaptive threshold with the global α-Rényi residuals are presented in Figure 14.

**Figure 15:**
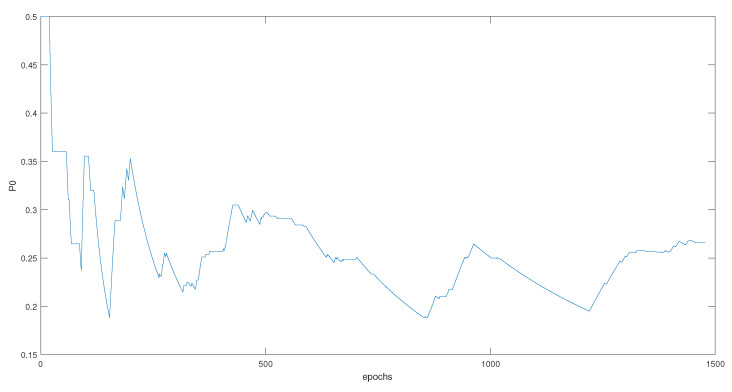
P0 during the whole trajectory.

The $P_{0}$ that is related to the previous threshold is seen in Figure 15.

The difference in behavior between the two $P_{0}$ and the two thresholds related to the fixed and balanced α is an indicator of a difference in the decision, which will surely affect the final estimation.

In the next section, we compare the final results and try to highlight the source of the differences.

#### 6.2.3. Results Comparison

**Figure 16:**
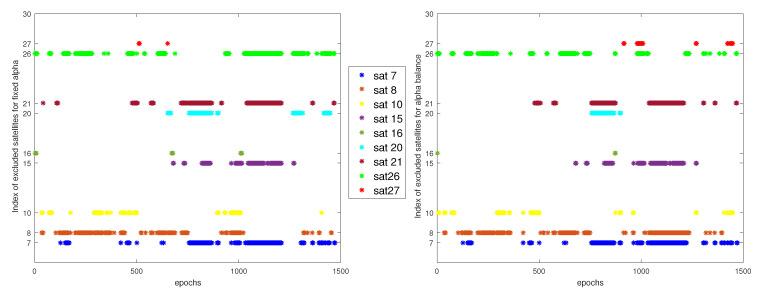
index of isolated satellites using fixed and balanced α.

We have to see the impact of the residuals design and the resulting decisions on the estimation position in order to compare the two scenarios. Let us first look at the effect of the two design architecture on the isolated measurements. Figure 16 illustrates the isolated satellites based on the decisions of the fixed and balanced α.

**Figure 17:**
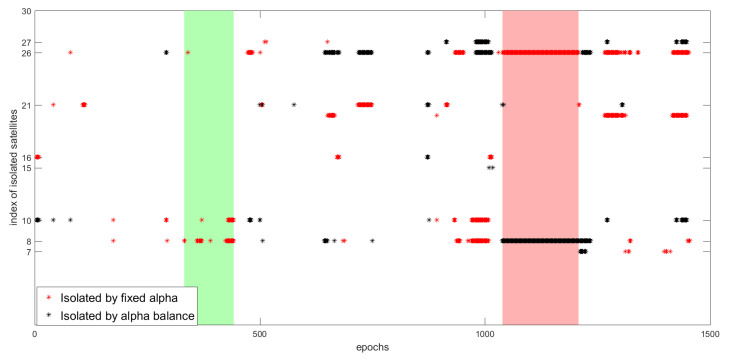
The difference in decisions between fixed α and α balance.

The two figures show a lot of commune measurements isolation, as well many differences that are present in different epochs and/or in the same epoch. Figure 17 only shows the differences between the isolated measurements in the two scenarios.

For example, in the fixed α scenario, the decision was to isolate satellite 26 from epoch 1039 to 1208 (pink zone). Where, in the α balance scenario, the satellite 26 is considered to be non-faulty and the decision was to isolate satellite 8. The same scenario is repeated between epochs 1424 and 1449 with two satellites in difference, and in many different epochs. On the other hand, the fixed α in many epochs decides to isolate some satellites, where, using α balance, no isolation occurred. For example, between epochs 332 and 442 (green zone), satellites 8 and 10 are isolated using the $RD_{0.5}$, and used in the final estimation by the α balance.

**Figure 18:**
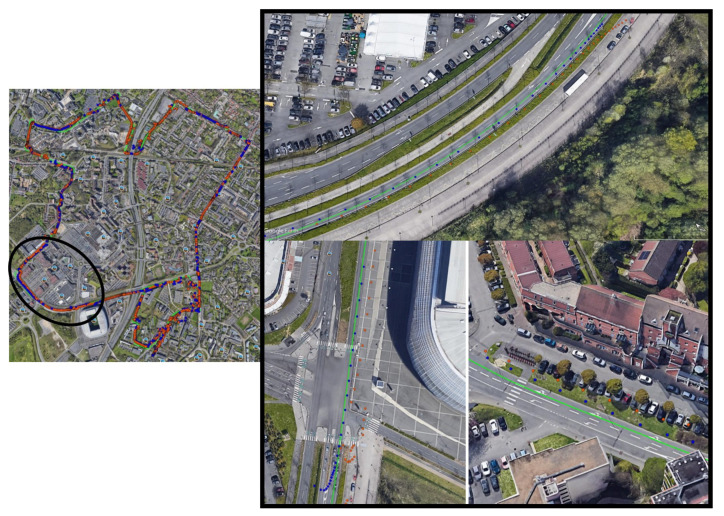
The effect of the decisions between fixed α and α balance on the position.

W will present the effect of these decisions on the final positioning estimation for both scenarios when compared to the reference using Figure 18 in order to judge fairly these decisions. Additionally, we zoomed on a selected area (black zone) to see clearly the effect of each decision on the position. Noting that the fixed α trajectory is plotted in orange color, and the blue trajectory refers to the α balanced estimated position.

**Figure 19:**
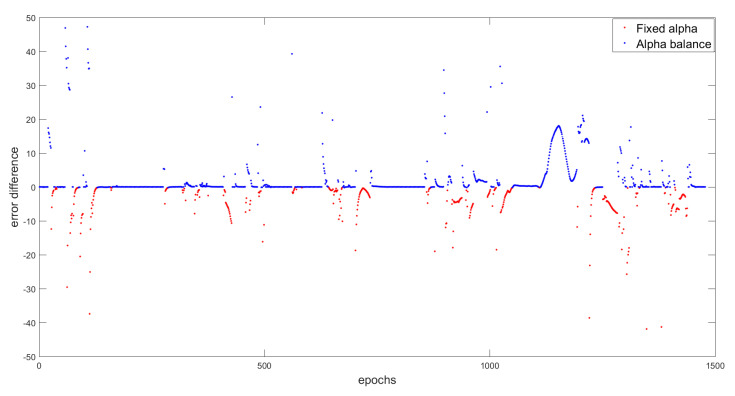
The difference in decisions between fixed α and α balance.

For deeper analysis, we can see, in Figure 19, the impact of the decisions for both scenarios on the position error calculated by the difference between the position error of fixed α diagnostic and the position error of the balanced α diagnostic. The blue color in the figure means that the balance α diagnostic at these epochs better estimates the position than the fixed α diagnostic, which is reflected by decreasing the position error. This difference, as we can see, can reach at some epochs the 50 m as difference. That difference means that the decision to use the observations at these epochs at the same weight as the evolution model was right and it can be seen as an error detection for the evolution model. Where, at the same epochs, the fixed alpha decisions were based on the fact that the evolution model is better than the observation model leading to a high error in the position estimation.

In contrast, the appearance of the red color is also logical. Because, in some epochs, giving the observations the same confidence as the prediction, under the bridges, for example, where the observations are distorted, is considered to be a mistake tbat will lead to an error estimation.

These results confirm our proposition to create a diagnostic layer that is able to take the changing environment into consideration and study each moment based on the new change and state. Hence, even if the results of the balanced α are better than the fixed α, but one can deny the fact of an adaptive α that is related to the changing environment and the changing of KPIs can be better.

As final results, and in order to evaluate the two diagnostic methods in numerical way and show the effect of the two FDI approaches on the position estimation, Table 2 presents the mean error, and the max error removed during the whole trajectory.

These results underline the performance of adaptive diagnosis between the two proposed scenarios. In addition, they show that it is not always a good policy to remove measurements from the final estimation and, to do so, adaptive diagnosis must be used while taking into account different factors, such as KPIs and environment. While, in some cases, adaptive diagnostics can act like normal diagnostics, such as when the vehicle is moving in the open sky over its entire trajectory. In this way, the adaptive diagnosis will provide a high degree of confidence in the observations at all times, and vice versa.

## 7. Conclusions

In this paper, the problem of fault-tolerant localization system for autonomous vehicle is treated. First, the concept of key performance indicators, which is well known in the domain of diagnostic, is introduced and the interaction between these terms is described. Subsequently, the adaptive fault tolerant fusion was presented. After explaining the relevance of the adaptive diagnostic concept, and how the fail-safe design architecture should be related to the changing environment of a vehicle, and able to provide the KPI requirements at each moment, the target to design an adaptive diagnostic layer through residual design was shown and detailed. The adaptive FTF approach was proposed using a parametric residuals that were generated through the α-Rényi divergence. To adapt this parameter α, we proposed a solution, called the α balance. This proposal led to a large number of statistical characterizations in order to create the faulty and non-faulty distributions. A solution is proposed to this problem using a mathematical model that is based on non-supervised real data. For decision-making through a thresholding method for the residuals, a new criterion design that was based on α-RD called α-Rényi criterion was developed. This criterion holds a large choice of criterion, where we linked the criterion to the corresponding calculated divergence. For the isolation part of the diagnostic, we showed the efficiency of using the unknown input observers as a separation method to isolate erroneous measurements. Finally, the whole algorithm was tested using real GNSS/odometer data using different tests trajectories. The results show good performance for the adaptive diagnostic, and the decision-making part.

The encouraging results obtained confirm our motivations for the choice of the methodological proposed tools, in particular the adaptive diagnostic in the informational framework. For the future research work, we consider the use of the INS with the GNSS/odo in tight coupling in order to reach higher level in accuracy and availability. In the diagnostic layer, the way for selecting the parameter α for residuals design in the adaptive diagnosis can be designed in integrating Artificial Intelligence (AI) tools. Regarding the decision part, the $P_{0}$ is calculated on the basis of the windowing method, which has some limitations regarding the fast adaptation to each case and the heuristic way of choosing the dimension of the window, which directly affects the sudden error detection. An AI tool is considered to be used for the $P_{0}$ estimation.

## Figures and Tables

**Table 1:** Data acquisition information for trajectory C3.

Trajectory Name	Acquisition Location	Number of Epochs	Trajectory Length
C3	Villeneuve-d’Ascq	1477	9844.71 m

**Table 2:** The type of errors removed.

Error Type in Meters	Error Removed by α Balance	Error Removed by Fixed α	Difference
Mean error	7.5022	6.5306	0.9716
Max error	57.0425	51.6895	5.3530

## Data Availability

Data is the property of CRIStAL Laboratory and cannot be shared publicly at this time.

## Abbreviations

The following abbreviations are used in this manuscript:

α-RD	α-Rényi Divergence
α-Rc	α-Rényi criterion
ADAS	Advanced Driver Assistance Systems
AFTF	Adaptive Fault-Tolerant Fusion
AI	Artificial Intelligence
CUSUM	CUmulative SUM
EKF	Extended Kalman Filter
FD	Fault Detection
FDI	Fault Detection and Isolation
FTF	Fault Tolerant Fusion
GNSS	Global Navigation Satellite System
IF	Informational Filter
IMU	Inertial Measurement Unit
INS	Integrated Navigation System
ITS	Intelligent Transportation System
KF	Kalman Filter
KL	Kullback-Leibler
KLC	Kullback-Leibler Criterion
KLD	Kullback-Leibler divergence
KPI	Key Performance Indicator
LGPR	Localizing Ground Penetrating Radar
MI	Mutual Information
NIF	Nonlinear Information Filter
NIS	Normalized Innovation Squared
NLOS	Non Line-Of-Sight
odo	Odometer
pdf	Probability density functions
PF	Particle Filter
QJSD	Quantum Jensen-Shannon divergence
SLAM	Simultaneous Localization And Mapping
SRR	Short Range Radar
SS	Separation Solutions
TBM	Transferable Belief Model
THR	Tolerable Hazardous Rate
UIO	Unknown Input Observers

## Appendix A

Based on Equation (10), and by considering that $\frac{\alpha }{2}{\left(X_{k/k-1}-X_{k/k}\right)}^{T}{\left(\sum_{\alpha }\right)}^{-1}\left(X_{k/k-1}-X_{k/k}\right)$ is the term *A*, and $-\frac{1}{2(\alpha -1)}\log\frac{|\sum_{\alpha }|}{|\sum_{k/k-1}{|}^{1-\alpha }{\left|\sum_{k/k}\right|}^{\alpha }}$ is the term *B*:

Term A can be written as:

(A1) $$\begin{array}{cc} \; & \frac{\alpha }{2}{\left(X_{k/k-1}-X_{k/k}\right)}^{T}{\left(\Sigma_{\alpha }\right)}^{-1}\left(X_{k/k-1}-X_{k/k}\right)\\ = & \frac{\alpha }{2}{\left(X_{k/k-1}-X_{k/k}\right)}^{T}\left(\frac{1}{\frac{\alpha }{Y_{k/k}}+\frac{1-\alpha }{Y_{k/k-1}}}\right)\left(X_{k/k-1}-X_{k/k}\right)\\ = & \frac{\alpha }{2}{\left(X_{k/k-1}-X_{k/k}\right)}^{T}\left(\frac{Y_{k/k-1}Y_{k/k}}{\alpha Y_{k/k-1}+\left(1-\alpha \right)Y_{k/k}}\right)\left(X_{k/k-1}-X_{k/k}\right) \end{array}$$

Term B can be written as:

(A2) $$\begin{array}{cc} \; & -\frac{1}{2(\alpha -1)}\log\frac{|\Sigma_{\alpha }|}{|\Sigma_{k/k-1}{|}^{1-\alpha }{\left|\Sigma_{k/k}\right|}^{\alpha }}\\ = & -\frac{1}{2(\alpha -1)}\log\left[\left|\frac{\alpha Y_{k/k-1}+\left(1-\alpha \right)Y_{k/k}}{Y_{k/k-1}Y_{k/k}}\right|\frac{1}{|\frac{1}{Y_{k/k-1}}{|}^{1-\alpha }{\left|\frac{1}{Y_{k/k}}\right|}^{\alpha }}\right]\\ = & -\frac{1}{2(\alpha -1)}\log\left|\frac{\alpha Y_{k/k-1}+\left(1-\alpha \right)Y_{k/k}}{Y_{k/k-1}Y_{k/k}}\right|-\frac{1}{2(\alpha -1)}\log\frac{|Y_{k/k}{|}^{\alpha }}{|Y_{k/k-1}{|}^{\alpha -1}}\\ = & \frac{1}{2(\alpha -1)}\log\left|\frac{Y_{k/k-1}Y_{k/k}}{\alpha Y_{k/k-1}+\left(1-\alpha \right)Y_{k/k}}\right|+\frac{1}{2(\alpha -1)}\log\frac{|Y_{k/k-1}{|}^{\alpha -1}}{|Y_{k/k}{|}^{\alpha }} \end{array}$$

## References

[1] National Highway Traffic Safety Administration (2015). Critical reasons for crashes investigated in the national motor vehicle crash causation survey. Wash. DC US Dep. Transp..

[2] Amini A., Vaghefi R.M., Jesus M., Buehrer R.M. Improving GPS-based vehicle positioning for intelligent transportation systems. Proceedings of the 2014 IEEE Intelligent Vehicles Symposium Proceedings.

[3] Jagadeesh G., Srikanthan T., Zhang X. (2004). A map matching method for GPS based real-time vehicle location. J. Navig..

[4] Brakatsoulas S., Pfoser D., Salas R., Wenk C. On map-matching vehicle tracking data. Proceedings of the 31st International Conference on Very Large Data Bases.

[5] Liu Y., Liu F., Gao Y., Zhao L. (2018). Implementation and analysis of tightly coupled global navigation satellite system precise point positioning/inertial navigation system (GNSS PPP/INS) with insufficient satellites for land vehicle navigation. Sensors.

[6] Kamijo S., Gu Y., Hsu L.T. (2015). Autonomous Vehicle Technologies: Localization and Mapping. IEICE ESS Fundam. Rev..

[7] Ward E., Folkesson J. Vehicle localization with low cost radar sensors. Proceedings of the 2016 IEEE Intelligent Vehicles Symposium (IV).

[8] Levinson J., Montemerlo M., Thrun S. (2007). Map-based precision vehicle localization in urban environments. Robot. Sci. Syst..

[9] Isermann R. (2006). Fault-Diagnosis Systems: An Introduction from Fault Detection to Fault Tolerance.

[10] Bader K., Lussier B., Schön W. (2017). A fault tolerant architecture for data fusion: A real application of Kalman filters for mobile robot localization. Robot. Auton. Syst..

[11] Ricquebourg V., Delafosse M., Delahoche L., Marhic B., Jolly-Desodt A., Menga D. Fault detection by combining redundant sensors: A conflict approach within the tbm framework. Proceedings of the COGIS’07.

[12] Shu-qing L., Sheng-xiu Z. A congeneric multi-sensor data fusion algorithm and its fault-tolerance. Proceedings of the 2010 International Conference on Computer Application and System Modeling (ICCASM 2010).

[13] Allerton D.J., Jia H. (2008). Distributed data fusion algorithms for inertial network systems. IET Radar, Sonar Navig..

[14] Jiang L. (2011). Sensor Fault Detection and Isolation Using System Dynamics Identification Techniques. Ph.D. Thesis.

[15] Mehra R.K., Peschon J. (1971). An innovations approach to fault detection and diagnosis in dynamic systems. Automatica.

[16] Sundvall P., Jensfelt P. Fault detection for mobile robots using redundant positioning systems. Proceedings of the 2006 IEEE International Conference on Robotics and Automation (ICRA).

[17] Morales Y., Takeuchi E., Tsubouchi T. Vehicle localization in outdoor woodland environments with sensor fault detection. Proceedings of the 2008 IEEE International Conference on Robotics and Automation.

[18] Ay N., Amari S.i. (2015). A novel approach to canonical divergences within information geometry. Entropy.

[19] Shannon C.E. (1948). A mathematical theory of communication. Bell Syst. Tech. J..

[20] Chen H.M., Varshney P.K., Arora M.K. (2003). Performance of mutual information similarity measure for registration of multitemporal remote sensing images. IEEE Trans. Geosci. Remote. Sens..

[21] Tmazirte N.A., El Najjar M.E., Al Hage J., Smaili C., Pomorski D. Fast multi fault detection & exclusion approach for GNSS integrity monitoring. Proceedings of the 17th International Conference on Information Fusion (FUSION).

[22] Mondal S., Chakraborty G., Bhattacharyya K. (2008). Robust unknown input observer for nonlinear systems and its application to fault detection and isolation. J. Dyn. Syst. Meas. Control..

[23] Bai L., Rossi L., Torsello A., Hancock E.R. (2015). A quantum Jensen–Shannon graph kernel for unattributed graphs. Pattern Recognit..

[24] Antolin J., Angulo J., Lopez-Rosa S. (2009). Fisher and jensen–shannon divergences: Quantitative comparisons among distributions. application to position and momentum atomic densities. J. Chem. Phys..

[25] Radhakrishnan C., Parthasarathy M., Jambulingam S., Byrnes T. (2016). Distribution of quantum coherence in multipartite systems. Phys. Rev. Lett..

[26] De Domenico M., Porter M.A., Arenas A. (2015). MuxViz: A tool for multilayer analysis and visualization of networks. J. Complex Netw..

[27] Al Hage J., El Najjar M.E., Pomorski D. (2017). Multi-sensor fusion approach with fault detection and exclusion based on the Kullback–Leibler Divergence: Application on collaborative multi-robot system. Inf. Fusion.

[28] Basseville M. (2013). Divergence measures for statistical data processing—An annotated bibliography. Signal Process..

[29] Joerger M., Chan F.C., Pervan B. (2014). Solution separation versus residual-based RAIM. Navig. J. Inst. Navig..

[30] Lewandowski W., Tisserand L. (2010). Relative characterization of GNSS receiver delays for GPS and GLONASS C/A codes in the L1 frequency band at the OP, SU, PTB and AOS. Bur. Int. Des Poids Mes. Tech. Rep..

[31] Histace A., Rousseau D. Divergence de Rényi comme mesure de contraste pour la détection d’objets dans des images bruitées. Proceedings of the GRETSI.

[32] Van Erven T., Harremos P. (2014). Rényi divergence and Kullback-Leibler divergence. IEEE Trans. Inf. Theory.

[33] Hobza T., Morales D., Pardo L. (2009). Rényi statistics for testing equality of autocorrelation coefficients. Stat. Methodol..

[34] Makkawi K., Ait-Tmazirte N., El Najjar M.E., Moubayed N. Combination of Maximum Correntropy Criterion & *α*-Rényi Divergence for a Robust and Fail-Safe Multi-Sensor Data Fusion. Proceedings of the 2020 IEEE International Conference on Multisensor Fusion and Integration for Intelligent Systems (MFI).

[35] Khoder M., Nourdine A.T., Nazih M. Fault Tolerant multi-sensor Data Fusion for vehicle localisation using Maximum Correntropy Unscented Information Filter and *α*-Rényi Divergence. Proceedings of the 2020 IEEE 23rd International Conference on Information Fusion (FUSION).

